# Retraction: Mouse-Induced Pluripotent Stem Cells Differentiate into Odontoblast-Like Cells with Induction of Altered Adhesive and Migratory Phenotype of Integrin

**DOI:** 10.1371/journal.pone.0224350

**Published:** 2019-10-21

**Authors:** The PLOS ONE Editors

After publication of this article [1] concerns came to light about the results reported in Fig 6:

- There are similarities between band shapes in several instances. For example, in panel B, β-tubulin bands are quite similar to one another in the E14 Tg2a blot; the β-tubulin (E14Tg2a) blot is similar to the β1 integrin (E14Tg2a) blot when aspect ratio is adjusted; and lane 3 of the α2 integrin (iPS) blot appears similar to lane 1 of the β-tubulin (E14Tg2a) blot when aspect ratio is adjusted.
- The blot and gel images have uniform black or white backgrounds that do not allow a confirmation of the integrity of these results as presented.

The authors notified the journal that the original data underlying the results in this article are not available.

Aichi Gakuin University is investigating this work. The investigating committee has tentatively confirmed that there are concerns about the quality of the western blot images reported in this article and that the underlying data are no longer available.

In light of the above concerns, the *PLOS ONE* Editors retract this article.

MM, RK, HY, KN, and HN agreed with retraction. NO and TH did not respond or could not be reached.
